# Emergency and successful management for a case of inferior vena cava perforation caused by cannulation of venovenous extracorporeal membrane oxygenation: A case report

**DOI:** 10.1097/MD.0000000000036399

**Published:** 2023-12-08

**Authors:** Xiangying Cen, Yanzhu Chen, Yi Chen

**Affiliations:** a Department of Intensive Care Medicine, Binhaiwan Central Hospital of Dongguan, Dongguan City, Guangdong Province, China; b The Key Laboratory for Prevention and Treatment of Critical Illness in Dongguan City, Dongguan City, Guangdong Province, China; c Department of Medical Intensive Care Medicine, The First Affiliated Hospital of Sun Yat-sen University, Guangzhou City, Guangdong Province, China.

**Keywords:** inferior vena cava, perforation, venovenous extracorporeal membrane oxygenation

## Abstract

**Rationale:**

Vascular complications associated with extracorporeal membrane oxygenation (ECMO) increase the in-hospital mortality. Perforation of the inferior vena cava (IVC) during venovenous extracorporeal membrane oxygenation (V-V ECMO) cannulation and subsequent emergency management prior to vascular surgery has rarely been reported.

**Patient concerns:**

A 72-year-old female was diagnosed with IVC perforation caused by venovenous extracorporeal membrane oxygenation cannulation.

**Diagnoses:**

Abdominal computed tomography venography with 3D reconstruction confirmed that the cannula tip had perforated the abdominal cavity from the conjunction of the iliac vein and IVC. As a result, the patient was diagnosed with inferior vena cava perforation.

**Interventions:**

Attempts to reposition the dislocated cannula using digital subtraction angiography were unsuccessful. However, we found that ECMO could maintain a stable blood flow; therefore, we decided to keep ECMO running, and to minimize blood loss from the puncture site, we ensured adequate blood transfusion while operating V-V ECMO. Subsequently, emergency laparotomy was performed to fix the vascular lesion, and we established a new V-V ECMO circuit through cannulation of the bilateral internal jugular veins.

**Outcomes:**

In the case of confirmed V-V ECMO-related vascular perforation of the IVC, it is crucial to continue ECMO device operation to maintain negative pressure in the IVC and position the dislocated catheter to block the perforation site, effectively controlling bleeding. Therefore, emergency laparotomy should be promptly performed for vascular repair. Fortunately, the patient recovered successfully and was subsequently discharged.

**Lessons:**

This case highlights several important lessons: When advancing a cannula, in this case, it is essential to first identify the guidewire placement to ensure proper guidance; In the event of a confirmed V-V ECMO-related vascular perforation of the IVC, maintaining negative pressure in the IVC through continued ECMO device operation and positioning the dislocated catheter to block the perforation site are crucial steps to control bleeding prior to emergency open vascular repair; After undergoing vascular repair, if ECMO support is still necessary, it is advisable to opt for a catheterization strategy that avoids previously repaired blood vessels.

## 1. Introduction

Extracorporeal membrane oxygenation (ECMO) is a medical technique used to provide cardiopulmonary support to critically ill patients. Venovenous extracorporeal membrane oxygenation (V-V ECMO) has been proven to be effective in treating severe respiratory failure caused by conditions such as severe acute respiratory distress syndrome, making it a widely used treatment for severe cases of influenza A^[1]^ and corona virus disease 2019.^[2]^ However, ECMO cannulation, the process of inserting tubes for ECMO, is a high-risk procedure with potential complications, including vascular complications. Common complications are thrombosis^[3]^ and obstruction^[4,5]^ of the inferior vena cava (IVC), which is the largest vein in the body. Although there have been reports of ECMO cannulation-related IVC perforation, there is limited information on the treatment of this complication. In a case report by Yeung Ng P et al, a patient with IVC anomaly experienced complications during ECMO cannulation, resulting in IVC perforation.^[6]^ Unfortunately, the report did not provide details on the treatment of the perforation, and the patient ultimately died of nosocomial pneumonia and septic shock. In this report, we present a case of IVC perforation caused by V-V ECMO cannulation and discuss the challenges faced during treatment. Our goal was to identify the potential causes of perforation and explore secure and efficient management strategies for similar incidents in the future.

## 2. Case history

**Figure 1.**
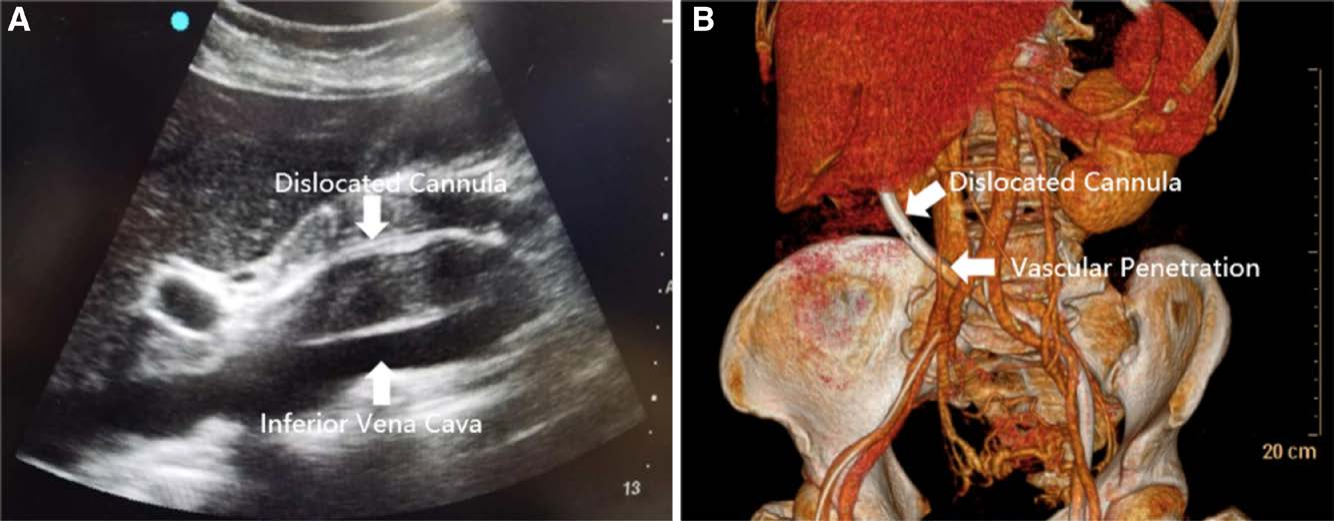
Abnormal position of the drainage cannula was detected upon imaging examinations. (A) Ultrasound showed that the cannula tip was not located at the entrance of the inferior vena cava in the right atrium, but close to the outer wall of the vessel. (B) Abdominal computed tomography venography (CTV) film with 3D reconstruction showing that the cannula tip perforated into the abdominal cavity from the conjunction of the iliac vein and inferior vena cava.

**Figure 2.**
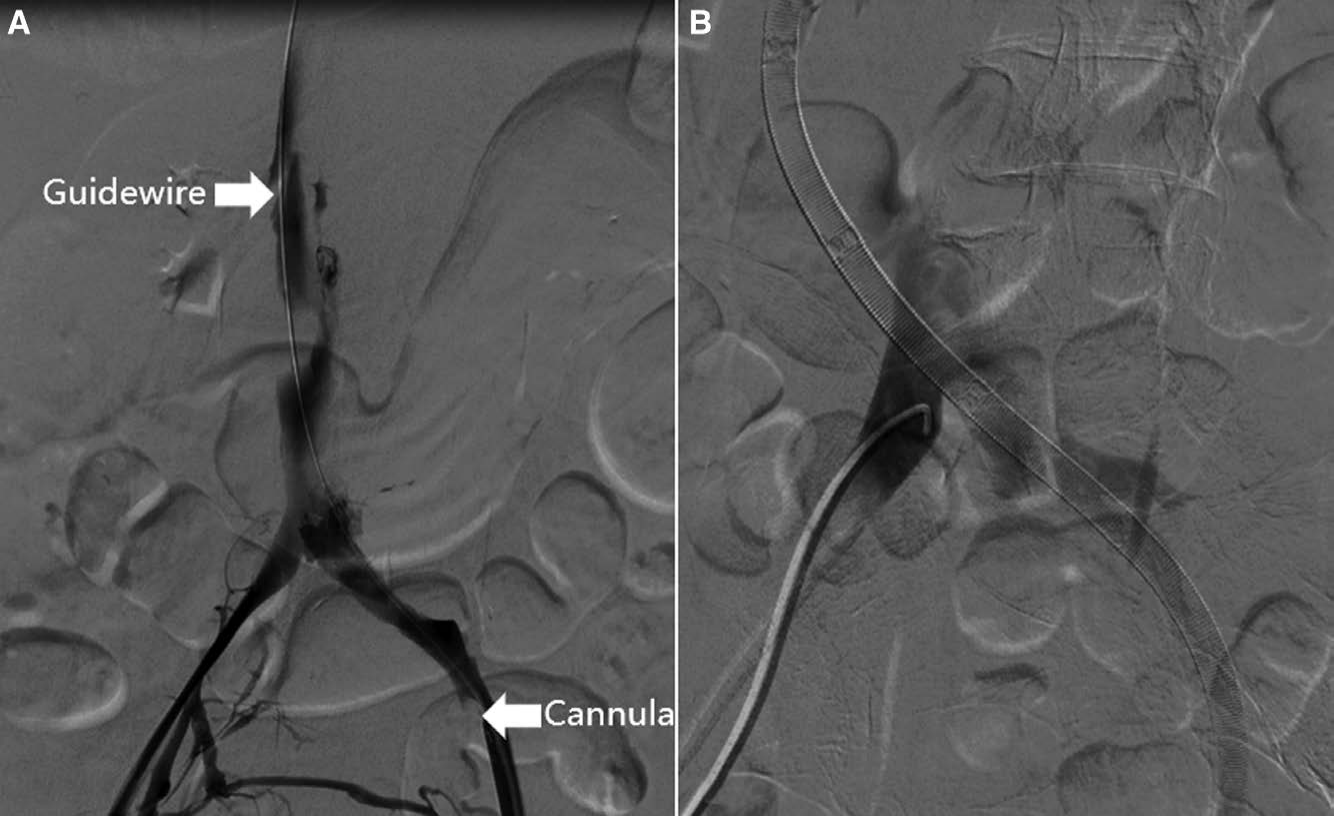
Findings under digital subtraction angiography. (A) The cannula tip could not be smoothly advanced along the guidewire in the conjunction of the iliac vein and IVC. (B) The cannula tip slipped out of the vessel again after the guidewire was retreated, blocking the breach. IVC = inferior vena cava.

**Figure 3.**
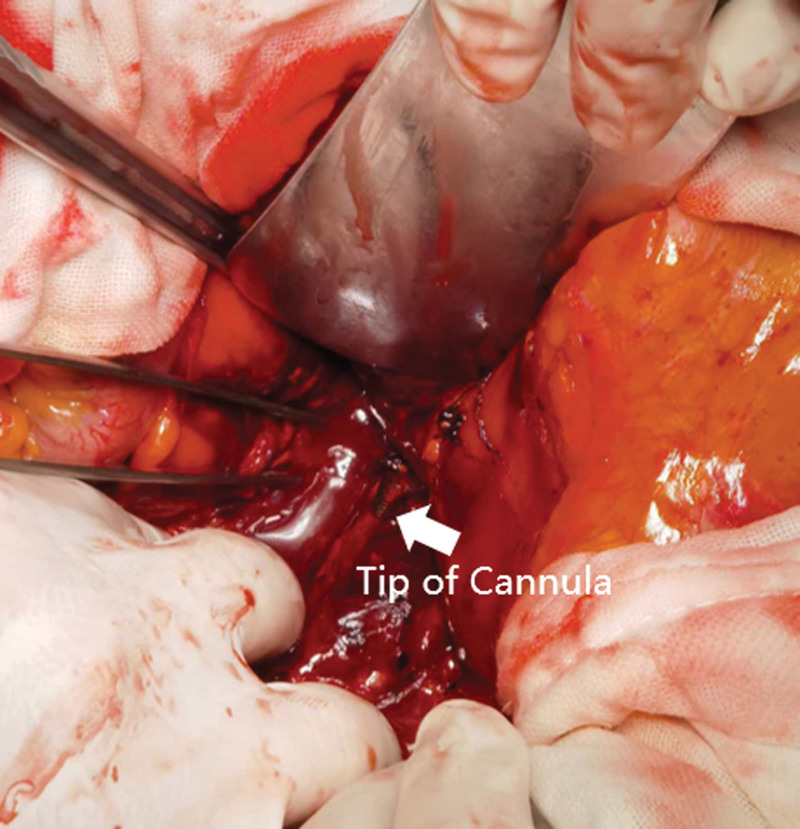
Finding in the laparotomy. The cannula tip penetrated from the conjunction of the iliac vein and inferior vena cava.

**Figure 4.**
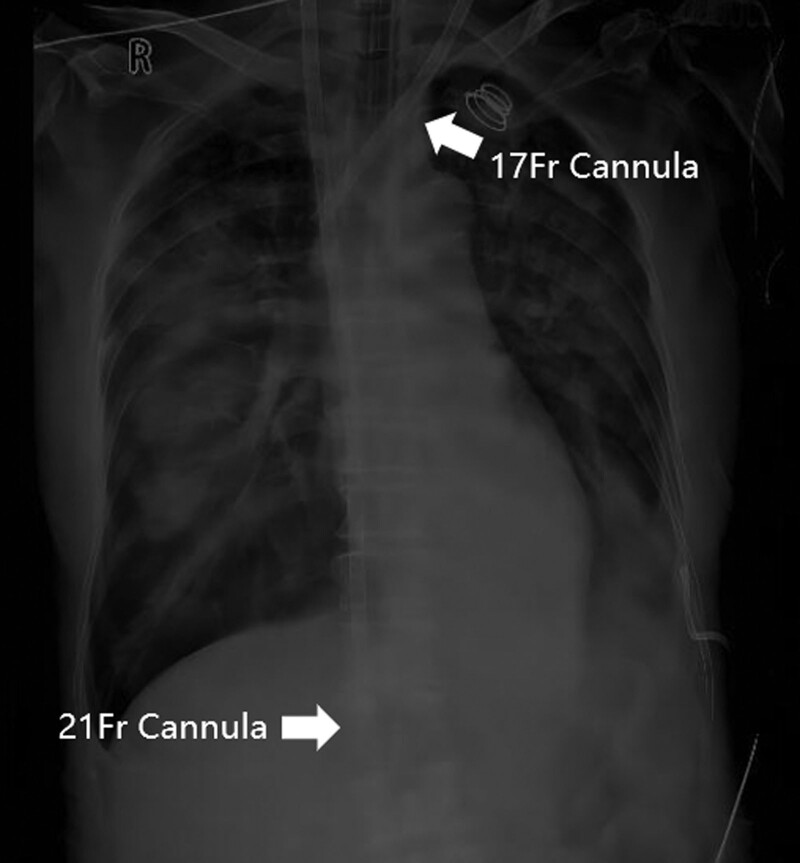
Ideal positions of the cannulas upon routine chest radiograph taken after second cannulation.

A 72-year-old female diagnosed with severe Chlamydia psittaci pneumonia-induced respiratory distress syndrome was admitted to our department. The patient had no relevant medical or family history. Despite lung-protective ventilation and prone position ventilation, the patient’s hypoxemia did not improve and worsened on day 7. Arterial blood gas analysis revealed hypoxygen (PaO_2_ 46.4 mm Hg, Positive end-expiratory pressure 12 cm H_2_O, Fraction of inspiration O_2_ 100%). V-V ECMO was initiated using point-of-care ultrasound-guided cannulation with the Seldinger technique. The left femoral vein (Maquet, Fr 21) and the right internal jugular vein (Maquet, Fr 17) were selected for catheterization. Following vascular puncture of the left femoral vein and placement of the guidewire, the drainage cannula was advanced along the guidewire until the patient suddenly presented with frequent ventricular premature beats. The operator quickly withdrew the guidewire and retained > 30 cm in the vascular lumen. The cannula continued to advance after the ventricular premature beats disappeared. When the insertion depth reached 28 cm, the operator felt slightly increased resistance and rotated the cannula mildly, eventually advancing the cannula smoothly to an insertion depth of 40 cm. After the ECMO circuit was connected, blood flow was initiated at 2.7 L/minutes and 3000 RPM. However, after 30 minutes, the blood flow dropped to 1.6 L/minute, accompanied by a decrease in the arterial blood pressure from 137/69 mm Hg to 110/58 mm Hg. Ultrasonography revealed that the cannula tip in the left femoral vein was not properly positioned at the entrance of the IVC in the right atrium. Instead, it was located close to the outer wall of the vessel (Fig. 1A). Emergency abdominal computed tomography venography confirmed the abnormal position of the drainage cannula, which had perforated the abdominal cavity from the conjunction of the iliac vein and IVC. Three pairs of side holes remained in the lumen of the iliac vein and IVC, maintaining on the cannula ECMO blood flow of 1.5 to 2.2 L/minutes and preventing excessive blood spilling (Fig. 1B). Considering the patient’s severe hypoxemia, we decided to continue ECMO support. Our initial plan was to withdraw the cannula tip to the endovascular lumen and then reposition it to the entrance of the IVC in the right atrium using digital subtraction angiography. However, the cannula tip could not pass through the conjunction of the iliac vein and IVC along the guidewire (Fig. 2A). Additionally, this attempt resulted in rapid massive bleeding, sequential hemorrhagic shock, and cardiac arrest, which forced us to stop the procedure. The cannula tip slipped out from the IVC when the guidewire was retracted, slowing the bleeding (Fig. 2B). After cardiopulmonary resuscitation and rapid blood transfusion, the patient recovered with stable hemodynamics. After an emergency multidisciplinary consultation in the operating room, we decided to continue ECMO and immobilize the dislocated catheter until the IVC could be intercepted in a laparotomy (Fig. 3). The cannula tip was identified as penetrating the junction of the iliac vein and inferior vena cava. ECMO was temporarily suspended, and oxygenation saturation was maintained at approximately 50% for 2 hours during the operation. After repairing the vascular damage, we planned to establish a new V-V ECMO circuit by catheterizing the internal jugular veins to avoid further damage to the IVC. However, the right internal jugular vein (Maquet, right internal jugular vein (Maquet, Fr 21) and left internal jugular vein (Maquet, Fr 17) were selected for cannulation. With guidance from transthoracic echocardiography, we placed the drainage cannula proximally from the suture of the IVC through the superior vena cava and the right atrium, establishing a new V-V ECMO circuit. The depths of cannula insertion on the right and left sides were 32 cm and 15 cm, respectively. Routine chest radiographs obtained after cannulation showed ideal positions for the cannulas (Fig. 4). During the V-V ECMO run, to monitor the patient’s brain function, we administered light sedation, daily awakening, and daily ultrasonic examinations of the bilateral internal jugular veins. The patient gradually improved and was weaned from ECMO and ventilator on days 14 and 17, respectively. She was transferred out of the ICU on day 29 and was discharged on day 45 without cerebral complications, stenosis, or thrombosis in the IVC.

## 3. Discussion

V-V ECMO can provide respiratory support for severe respiratory failure, making the use of V-V ECMO dramatically increasing over the years, especially after the outbreak of coronavirus disease 2019.^[7]^ However, V-V ECMO can lead to various complications, including circuit-related mechanical issues, renal impairment, cardiovascular compromise, and hemorrhage, while cannula-related complications account for 6.6% of complications.^[7]^ Vascular complications can result in a 24% increase in the median hospital charge and a 34% increase in in-hospital mortality.^[8,9]^ Common cardiovascular perforations due to ECMO cannulation include cardiac tamponade^[10]^ and superior vena cava perforation,^[11]^ with a high incidence in neonates and children.^[6,12]^ However, IVC perforation in adults and its specific treatment are rarely documented.^[13]^

In the present case, vascular perforation occurred during cannulation and was an iatrogenic complication. We did not reconfirm the position of the guidewire with ultrasound before advancing the cannula again, because we believed that the length of the guidewire in the vascular lumen was up to 50 cm. However, the length of the guidewire was shorter than we thought, as the drainage cannula penetrated from the conjunction of the iliac vein and inferior vena cava. We speculate that during arrhythmia, the tip of the guidewire was retracted into the cannula, and when the cannula was advanced again without sufficient guidewire guidance, the cannula tip penetrated the IVC.

After confirming vascular perforation, our initial attempt to replace the dislocated cannula through digital substraction angiography failed and worsened the situation. The complex treatment process highlights the importance of continuously operating the ECMO device and immobilizing the dislocated catheter before performing emergency laparotomy. These measures create negative pressure in the IVC and minimize bleeding from the vascular perforation.

It is generally not recommended to establish a V-V ECMO circuit via the bilateral internal jugular veins. Inserting a drainage cannula into the right internal jugular vein can reverse the blood flow path.^[7]^ Additionally, cannulation in the bilateral internal jugular veins may affect the patency of cerebral venous flow and lead to catastrophic cerebral events.^[14]^ In our case, the patient’s bilateral femoral vein vessels were not suitable for catheterization, and a dual-lumen bicaval cannula was unavailable; therefore, we chose the bilateral internal jugular vein to establish a V-V ECMO circuit and obtain a good therapeutic effect. When choosing the bilateral internal jugular cannula insertion sites, we must pay close attention to preventing thrombosis and recirculation and monitoring brain function during management.

## 4. Conclusion

V-V ECMO is a valuable treatment option for severe respiratory failure. However, it is important to be aware of the potential risks associated with cannulation, as vascular complications can be life threatening and pose a challenge in terms of management. We report an iatrogenic IVC perforation arising from V-V ECMO cannulation, which highlights several important lessons: When inserting a cannula, it is crucial to ensure the proper placement of the guidewire to accurately guide the procedure. This step helps minimize the risk of complications; In the event of a confirmed V-V ECMO-related vascular perforation of the IVC, maintaining negative pressure in the IVC through continued ECMO device operation and stabilizing the dislocated catheter to block the perforation site are crucial steps to control bleeding prior to emergency open vascular repair; After undergoing vascular repair, if ECMO support is still necessary, it is advisable to opt for a catheterization strategy that avoids the previously repaired blood vessels. Establishing a V-V ECMO circuit via cannulaton in bilateral internal jugular veins requires comprehensive cannulation plan, and precise monitoring during ECMO run. This approach reduces the risk of reinjury, and ensures safer and more effective in ECMO support. By implementing these lessons, healthcare providers can enhance patient safety and outcomes when utilizing V-V ECMO for respiratory support.

## Author contributions

**Writing – original draft:** Xiangying Cen.

**Writing – review & editing:** Yanzhu Chen, Yi Chen.
